# Development of an Extended-Reality (XR)-Based Intervention to Treat Adolescent Obesity

**DOI:** 10.3390/ijerph19074264

**Published:** 2022-04-02

**Authors:** Neal Malik, Wagner L. Prado, Sara Lappan, Mihaela Popescu, Bryan Haddock, James O. Hill

**Affiliations:** 1 Department of Health Science and Human Ecology, California State University, San Bernardino, CA 92407, USA; 2 Department of Kinesiology, California State University, San Bernardino, CA 92407, USA; wagner.prado@csusb.edu (W.L.P.); bhaddock@csusb.edu (B.H.); 3 Couples and Family Therapy Program, Alliant International University, Alhambra, CA 91803, USA; sara.lappan@alliant.edu; 4 Department of Communication Studies, California State University, San Bernardino, CA 92407, USA; popescum@csusb.edu; 5 Department of Nutrition Sciences, University of Alabama at Birmingham, Birmingham, AL 35233, USA; hillj@uab.edu

**Keywords:** obesity, adolescence, virtual reality, extended reality

## Abstract

Public health policies aimed at obesity reduction are more often directed toward adults than children. This is alarming given that rates of childhood obesity have been steadily increasing, and, if not treated early, adolescents with obesity may develop comorbidities into adulthood. Lifestyle-based interventions are the cornerstone of childhood obesity treatment. Recently, extended-reality (XR)-based interventions have been incorporated into the treatment of obesity, and parents and adolescents perceive virtual reality (VR) interventions as a promising approach to increasing physical activity levels and improving eating habits. VR is a tool that fits perfectly with contemporary adolescent culture, which is radically different from that of just two generations ago. It is plausible that an XR-based intervention for treating adolescents with obesity could have a profound influence on obesity management over the long-term. An understanding of adolescents’ preferences, wants, and needs must be considered in the development of new interventions. We suggest that VR interventions can provide a new approach to weight management for children and adolescents and provide recommendations to assess adolescents’, caregivers’, and primary care providers’ needs. These needs could then be used for the development of an XR-based intervention aimed at inducing sustained lifestyle changes in adolescents with obesity.

## 1. Introduction: Time for a Change?

Multidisciplinary behavioral interventions are considered the most effective approach for sustainable behavior change to reduce childhood obesity [1].

**Figure 1:**
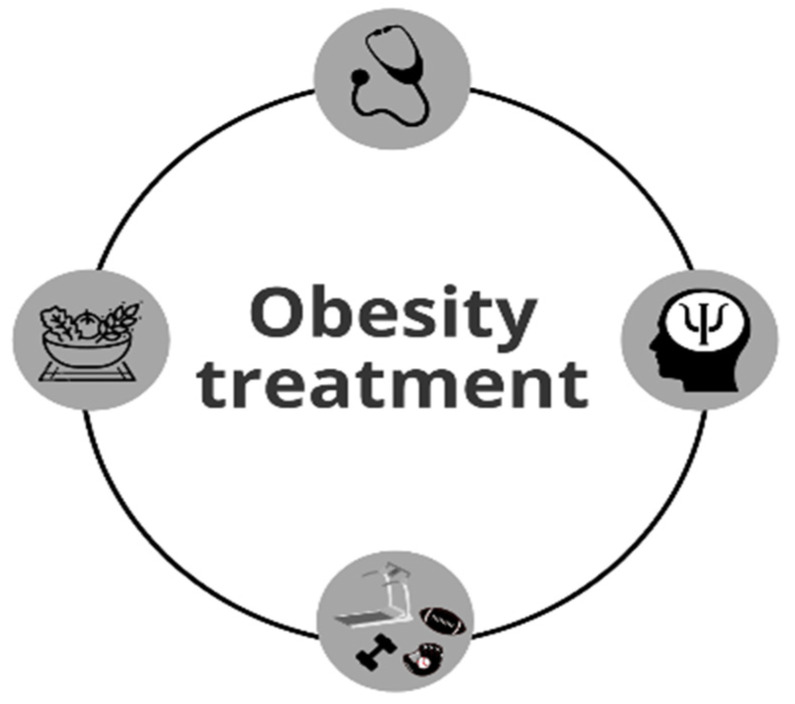
Standard multidisciplinary obesity treatment composed of medical and nutritional therapies along with psychological counseling and physical activity programs.

However, given that the incidence of adolescent obesity continues to increase [2], it is plausible that the traditional model for obesity treatment (Figure 1) is not ideal [3]. Commonly reported barriers to long-term adherence to weight management programs include the high time commitment and safety concerns [4]. These commonly reported barriers consequently result in poor treatment adherence, low retention rates [5], and poor long-term success rates. Our prior research examining the impact of inpatient behavioral interventions on health-related outcomes among adolescents with obesity found high dropout rates ranging from 45% to 55% [6,7,8,9,10,11]. Other studies have found that outpatient interventions may increase program adherence [12,13,14,15]. Technology-based approaches may help keep participants engaged in behavioral interventions [16]. Internet-based technologies developed with feedback from patients and primary care providers could help reduce barriers to face-to-face interventions [17], e.g., cost, travel, and scheduling. Going further, extended reality (XR) technologies such as augmented (AR), virtual (VR), and mixed reality (MR) could substantially increase participant engagement and hold the promise of revolutionizing disease prevention approaches for children.

Extended reality (“XR”) is an umbrella term for a range of technologies that blend physical and digital environments. Depending on the level of immersion they allow the participants, these technologies are situated in a “reality–virtuality continuum” [18]. At one end of this continuum, augmented reality (AR) stands for technologies that simply superimpose digital information (e.g., text, multimedia constructs, and haptic information) onto the real environment, effectively turning it into a digital interface accessible through mobile devices. At the other end of the continuum, virtual reality (VR), accessible through specialized head-mounted displays, provides a digitally constructed environment that perceptually surrounds the participant and allows real-time interactions from a first-person perspective. Mixed reality (MR) technologies, located in the middle of the continuum, use specialized glasses to recreate the real environment in an enhanced holographic display that the participant can manipulate. Here, we propose a VR intervention which would allow for participants to engage in a virtual space with the ability to manipulate the environment.

Approximately 1% of scientific publications have focused on the health of adolescents [19].

**Figure 2:**
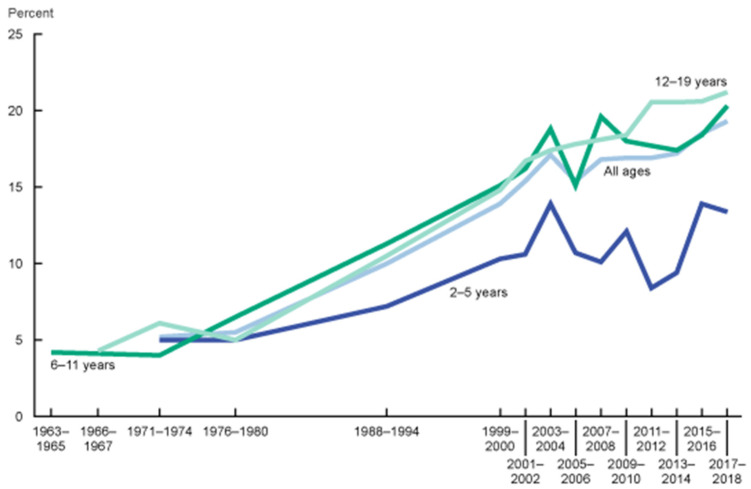
Trends in obesity prevalence in the U.S. (1963–2018). CDC. National Health and Nutrition Examination Survey.

This is problematic, as overweight adolescents are more likely to become overweight adults, and obesity in adolescence is associated with an increased risk of multiple comorbidities in adulthood, even if obesity does not persist [20]. Data from the 2017–2018 National Health and Nutrition Examination Survey (NHANES) revealed that 21.2% (+1.3) of US adolescents aged 12 to 19 years are obese, and these rates have been steadily increasing (Figure 2) [2].

Behavioral lifestyle-based interventions are the gold standard of adolescent obesity treatment [21]. In fact, we previously demonstrated that several types of in-person lifestyle interventions aiming to improve eating behavior, mental health, and physical activity levels were equally effective for inducing short-term benefits in several health-related outcomes, including quality of life in adolescents with obesity [6,7,8,9,10,11,22,23]. Unfortunately, the magnitude of the effects on these key outcomes was small, and the interventions appear ineffective in the long-term.

This manuscript’s overarching objective is to encourage the development and evaluation of XR technology use in lifestyle interventions. We provide recommendations to assess adolescents’, caregivers’, and primary care providers’ needs to be used for the development of an XR-based intervention aimed at inducing sustained lifestyle changes in adolescents with obesity.

## 2. Incorporating Digital Technologies into Weight Management Interventions

Digital technologies and extended reality (XR) have been incorporated into the treatment of several medical conditions. A recent systematic review [17] suggested that technology-based interventions may increase weight loss and improve treatment adherence among adults with obesity. Previous research has shown that wearable trackers (e.g., Fitbits) may increase physical activity levels and assist with weight management in adults [24]. In adolescents, however, the use of wearable tracking devices was only shown to be beneficial in the short-term [25]. Within the broader technology-based spectrum, activity-monitoring devices (e.g., Fitbits) have ease-of-wear utility and allow for tracking health-related behaviors but lack the ability to offer a deep and immersive virtual experience. XR could potentially incorporate health behavior tracking while providing an enriched experience.

### XR vs. VR

Thanks to their unique media affordances, XR technologies could potentially enable a paradigm shift in obesity interventions. Because of their high resolution, wide field-of-view, and, in the case of VR, head-tracked display with real-time motion capture, XR environments support the perceptual illusion of “being there” (presence), as well as sensorimotor and affective reactions in response to this perception [26,27,28,29]. Presence and emotional engagement in VR environments have been shown to correlate with health self-efficacy [30,31] and motivation to engage in healthy behaviors [32,33]. Additionally, XR environments permit the integration of different forms of treatment (visual–motor, cognitive, and behavioral) into various forms of therapy. In combination with presence and immersion, this multimodal treatment integration has been shown to be successful in addressing the etiology of obesity, especially in regard to eating disorders and negative body image [34,35,36]. However, no consensus has been reached regarding which forms of XR intervention are the most effective over the long-term, although one study demonstrated that VR-enhanced treatments may increase adult patients’ likelihood of maintaining or improving weight loss one year after treatment [36]. XR is a powerful enhancement that can be integrated with other technology- (e.g., Fitbit trackers and nutrient analysis applications) and non-technology-based methods (e.g., psychological counseling) to help effectuate the significant lifestyle changes necessary for successful obesity mitigation and reduction. It is a tool that is, itself, technologically coming of age. It is a tool whose attributes fit perfectly with contemporary adolescent culture, which is radically different from that of just two generations ago. Additionally, given that adolescent obesity is often attributed to the excessive use of technology, we may be able to use technology as a means to reduce this very problem [37,38].

## 3. Are Digital Technologies Effective for Weight Management?

Horne and Cols (2020) reported a positive impact of electronic avatars on weight loss and motivation in adults with obesity [39]. However, both systematic reviews were conducted among the adult population, and data related to childhood and adolescent populations are scarce. Traditionally, gaming may have been viewed as a sedentary activity, whereas both parents and adolescents in a healthy weight range now perceive and describe virtual reality interventions as a promising approach for increasing physical activity levels [40,41]. Gamification, the use of game design principles to influence socially significant human behavior [42], has been incorporated as a way to modify health behaviors such as dietary habits and physical activity [43,44]. When technological gamification was used as a means to promote weight management among school-aged children, researchers discovered that this method was effective at reducing children’s BMI [45]. Taking this into consideration, it is plausible that an XR-based intervention for treating adolescent obesity could have a profound influence on obesity management over the long-term.

Parents and caregivers play a pivotal role in facilitating and stimulating adolescents’ engagement with and compliance to health behaviors [46]. Health professionals also play a critical role by prescribing appropriate interventions for their patients. Therefore, in order to develop an effective XR-based intervention, both groups must be involved in its development. Of note, traditional behavioral interventions for treating adolescent obesity were developed based on biological-centered models and adapted from data gathered in the adult population. Therefore, these data may not be generalizable to adolescents with obesity. An understanding of adolescents’ preferences, wants, and needs must be considered in the development of new interventions, as proposed here.

Incorporating a video game component into a traditional exercise session has been shown to increase energy expenditure in children (7–14 years of age) without an increase in perceived exertion [47]. These data support the idea that the wants and needs of adolescents with obesity may play a central role in successful treatment. We recently demonstrated that a less intensive (fewer contact hours) behavioral counseling intervention for the management of adolescent obesity, based on adolescents’ personal motivation and autonomy, is effective for improving psychological outcomes (such as depression, anxiety, and eating disorders), as well as for inducing changes in body composition after 24 weeks of intervention [48]. Of note, adherence and compliance to such interventions are higher among those engaged in recreational physical activity programs, and even higher in boys. This suggests gender differences may exist regarding treatment preferences among adolescents, and this will therefore be addressed as part of our study.

We also conducted interviews with low-income, single, female caregivers to discuss both risk and protective factors associated with childhood obesity. Caregivers’ suggestions for future interventions aimed at addressing childhood obesity were also assessed [49,50]. Using the Socioecological Framework [51] as a guide, thematic analysis was used to analyze the interviews. Participants cited the financial cost of recreation as a barrier to their access and participation. This can be addressed with the currently proposed study. In terms of suggestions made by the participants for future obesity interventions, participants cited transportation and childcare as barriers to retention in programming. Media integration and modifications to increase participation were also suggested. Participants also reported a desire to incorporate health professionals as part of the intervention process. These recommendations are addressed in the current proposal in the following ways: reducing commonly reported barriers, increasing intervention accessibility, integrating extended reality into the programming, and grounding the intervention by systematically gathering the input of participants, caregivers, and health professionals.

## 4. Theoretical Underpinnings

We propose Community-Based Participatory Research (CBPR) as the philosophical framework to guide the study design for incorporating XR technology into weight management programs.

CBPR, in which academia and the community form a partnership to address community issues, and in which the community is integral to all phases, has been found to successfully address health disparities [52,53] and results in demonstrably positive health outcomes [54]. Community input enhances the quality and acceptability of interventions [55]. In keeping with CBPR principles, a community advisory group (CAG) would guide all steps of the study. Researchers would report to the CAG before and after each step of the study, and in turn, the CAG provides guidance to the study team.

Fraenkel’s [55] participatory model of intervention and evaluation would serve as the guideline. This model aligns with CBPR and emphasizes a collaborative approach that equitably includes community partners at all stages of the research process and would serve as the ethical guide. Ultimately, this model consists of eight sequential steps: (1) engagement with the community and the formation of collaborative relationships, (2) intensive interviewing of potential participants and community leaders, (3) qualitative data analysis with members from the community, (4) the creation of program formats and program manuals, (5) the implementation of a pilot study and session-by-session evaluation by participants, (6) interviewing participants regarding each cycle of the intervention, (7) testing for efficacy, and (8) adaptation to larger settings and dissemination. This process ensures that the intervention is developed from the ground up, while incorporating community input.

## 5. Mapping Implementation of Digital Technologies for Weight Management

Phase 1: Community Collaborators. Collaborators within the community would form the CAG. Ideally, it would consist of 8 to 10 members of the community, potential intervention participants, and local community stakeholders. Additionally, the CAG members from each aforementioned group would also participate in the focus groups (see Phase 2: Focus Groups below). The CAG would involve adolescents, caregivers of adolescents, primary care providers, and health professionals/researchers within the fields of psychology, kinesiology, public health, and nutrition (with each group represented). The CAG members would provide feedback on every aspect of the protocol including the consent form, the focus group protocol, recruitment materials, and recruitment methodology. The CAG members would also serve as the primary referral source for participant recruitment.

Multiple sampling methods would be used to recruit CAG participants. However, recruitment through health care professionals’ referrals (e.g., adolescents’ primary care physicians) and outpatient clinics (e.g., diabetes outpatient care, adolescent weight management programs, support groups, etc.) may be ideal.

Phase 2: Focus Groups. Focus groups have been established as an effective mechanism for eliciting opinions, perspectives, and beliefs about sensitive topics, especially in multicultural settings, and for providing insights that cannot typically be gleaned using more structured research methods [52,53,56]. We propose conducting four focus groups with each group representing a different invested party. Group one will contain female-identified adolescents, group two will contain male-identified adolescents, group three will contain caregivers of adolescents, and group four will contain professionals and researchers within the fields of psychology, kinesiology, public health, and nutrition. Purposive sampling can be used to ensure each group consists of a varied population sample, which is vital to developing a thorough understanding of extended reality (XR) and its applicability to weight management among adolescents. The purpose of the focus groups is to investigate and understand participants’ attitudes toward and expectations of XR technology treatment for adolescent weight management. Therefore, participant diversity (i.e., participants with differing identities, professions, and ages as achieved through the four different focus groups) should be emphasized in order to provide a broad range of perspectives.

As per focus group methodology, focus groups will continue to be scheduled until the data reach a saturation point, that is, with no new themes emerging [57]. Based on past experience, we anticipate being able to reach data saturation with three sessions for each group [57,58].

Phase 3: Creation of an XR Prototype. Themes that have emerged from data analyses in Phase 2 would be reported to a technological design team. This team would then develop an XR prototype. This prototype would then be presented to the CAG and focus group members for feedback. Focus groups would continue to be scheduled until the data reach a saturation point. These data would then be collected, analyzed, and presented to the technological design team to guide modifications to the platform. These steps would be repeated until the CAG reports acceptance of the prototype.

### Challenges and Opportunities

There is significant evidence that females speak less in group settings when males are present [59]. Additionally, among teens, single-gender focus groups that lack familiarity with one another and whose ages differ by less than 2 years may encourage a diversity of opinions and help elicit fruitful conversations [60]. It may be important to ensure that the moderator for each group shares the same gender identity as the participants so as to not limit member participation or potentially skew the focus group discussion [60]. Additionally, conducting separate focus groups for adolescents and their caregivers would help to elicit honest, unfiltered responses.

## 6. Conclusions

There is an urgent need to improve lifestyle modification programs to help reduce obesity in children and adolescents. We propose that XR technologies may be able to significantly improve adherence to behavioral interventions, and we challenge researchers to explore XR-based intervention in treating adolescent obesity, given it could have a profound influence on obesity management over the long-term.

## Data Availability

Not applicable.
